# The readiness and motivation interview for families (RMI-Family) managing pediatric obesity: study protocol

**DOI:** 10.1186/s12913-017-2201-8

**Published:** 2017-04-11

**Authors:** Geoff D. C. Ball, Nicholas D. Spence, Nadia E. Browne, Kathleen O’Connor, Suja Srikameswaran, Joanna Zelichowska, Josephine Ho, Rebecca Gokiert, Louise C. Mâsse, Valerie Carson, Katherine M. Morrison, Jennifer L. Kuk, Nicholas L. Holt, Maryam Kebbe, Nicole D. Gehring, Melody Cesar, Heidi Virtanen, Josie Geller

**Affiliations:** 1 Department of Pediatrics, Faculty of Medicine and Dentistry, University of Alberta, Edmonton, AB Canada; 2 Department of Psychiatry, University of British Columbia, Vancouver, BC Canada; 3 Department of Pediatrics, Cumming School of Medicine, University of Calgary, Calgary, AB Canada; 4 Faculty of Extension, University of Alberta, Edmonton, AB Canada; 5 BC Children’s Hospital Research Institute, School of Population and Public Health, Faculty of Medicine, University of British Columbia, Vancouver, BC Canada; 6 Faculty of Physical Education and Recreation, University of Alberta, Edmonton, AB Canada; 7 Department of Pediatrics, Faculty of Health Sciences, McMaster University, Hamilton, ON Canada; 8 Faculty of Health, School of Kinesiology and Health Science, York University, Toronto, ON Canada

**Keywords:** Obesity, Pediatric, Parent, Family, Motivational interviewing, Lifestyle, Health behaviour

## Abstract

**Background:**

Experts recommend that clinicians assess motivational factors before initiating care for pediatric obesity. Currently, there are no well-established clinical tools available for assessing motivation in youth with obesity or their families. This represents an important gap in knowledge since motivation-related information may shed light on which patients might fail to complete treatment programs. Our study was designed to evaluate the measurement properties and utility of the Readiness and Motivational Interview for Families (RMI-Family), a structured interview that utilizes a motivational interviewing approach to *(i)* assess motivational factors in youth and their parents, and *(ii)* examine the degree to which motivation and motivation-related concordance between youth and parents are related to making changes to lifestyle habits for managing obesity in youth.

**Methods:**

From 2016 to 2020, this prospective study will include youth with obesity (body mass index [BMI] ≥97th percentile; 13–17 years old; *n* = 250) and their parents (*n* = 250). The study will be conducted at two primary-level, multidisciplinary obesity management clinics based at children’s hospitals in Alberta, Canada. Participants will be recruited and enrolled after referral to these clinics, but prior to initiating clinical care. Each youth and their parent will complete the RMI-Family (~1.5 h) at baseline, and 6- and 12-months post-baseline. Individual (i.e., youth or parent) and family-level (i.e., across youth and parent) responses to interview questions will be scored, as will aspects of interview administration (e.g., fidelity to motivational interviewing tenets). The RMI-Family will also be examined for test-retest reliability. Youth data collected at each time point will include demography, anthropometry, lifestyle habits, psychosocial functioning, and health services utilization. Cross-sectional and longitudinal associations between individual and family-level interview scores on the RMI-Family and these clinical measures will be examined.

**Discussion:**

As a measurement tool drawing on family-centered care and motivational interviewing, the RMI-Family was designed to increase understanding of the role of motivational factors in pediatric obesity management, allowing healthcare providers and policymakers to manage pediatric obesity more effectively and efficiently. Findings will help to create an innovative, tailored model of health care delivery that uses resources judiciously and is designed to best meet families’ needs.

**Electronic supplementary material:**

The online version of this article (doi:10.1186/s12913-017-2201-8) contains supplementary material, which is available to authorized users.

## Background

Approximately 1.5 million children in Canada meet the definition of overweight or obese [1]. Pediatric obesity usually persists into adulthood [2] and can lead to adverse metabolic health outcomes, including type 2 diabetes, hypertension, and cardiovascular disease [3]. Overall, the data are compelling and unequivocal: pediatric obesity is a prevalent and chronic condition, so innovative approaches are required to prevent and manage health risks associated with obesity in order to optimize quantity and quality of life [4].

Family motivation plays a vital role in treatment engagement, and ultimately in the successful management of pediatric obesity. Expert guidelines encourage clinicians to assess motivational factors (e.g., perceived importance of lifestyle changes and competence in executing them) within a comprehensive assessment prior to initiating treatment [5, 6]. Currently-available tools measure motivational factors in youth and parents independently [7, 8]; however, to our knowledge, there are no clinical tools available for determining motivation at the family level, a factor that may play a substantial role in addition to (and perhaps beyond) motivation at the individual level.

To address this limitation, the *Readiness and Motivation Interview for Families* (RMI-Family) was created as a structured interview for assessing youth and parent motivation as well as agreement among them in regards to making lifestyle changes in pediatric weight management [9]. The RMI-Family was modelled on the *Readiness and Motivation Interview* (RMI) that was developed originally for use in the management of eating disorders (e.g., anorexia nervosa) and emerged as a ‘gold standard’ approach to assessing treatment motivation in that population [10–12]. The RMI-Family was based on the tenets of motivational interviewing (MI), a directive, client-centered, and empirically-supported clinical approach to helping individuals resolve ambivalence about change [13] across a range of lifestyle behaviours, including smoking [14], substance abuse [15], as well as nutrition and physical activity [16]. We recently reported that the RMI-Family was feasible for administration in a clinical setting and was easily understood by families [9]. As a logical next step to gain insight into both treatment and clinical outcomes, the current study was designed to evaluate the effectiveness of the RMI-Family. We will *(i)* examine the reliability and validity of the RMI-Family as a measure of youth and parent motivation to change nutrition and physical activity habits as well as concordance in motivation among these family members to *(ii)* investigate the extent to which youth and parents presenting for treatment of pediatric obesity express motivation to change, as well as concordance across family members on this construct, and *(iii)* examine whether individual and/or family-level motivation for change at baseline predicts clinical and treatment outcomes, including nutrition and physical activity habits, weight management, health services utilization, and program attrition at 6- and 12-months follow-up.

## Methods

### Study design

Our project is a prospective observational study involving youth with obesity (13 – 17 years old) and their parents (primary caregivers) that will be conducted from 2016 to 2020 at two primary obesity management clinics in Alberta, Canada (Stollery Children’s Hospital, Edmonton, AB; Alberta Children’s Hospital, Calgary, AB).

### Study participants

We aim to recruit a total of 250 family dyads (one youth and one parent^1^), for a total of 500 participants. Each study site will recruit 125 families into the study, with an equal ratio of boys-to-girls to enable gender comparisons. Our sample size calculations were performed using G*Power [17]. Sample size estimation was calculated based on the analyses requiring the greatest sensitivity (i.e., links between individual and family-level scores on the RMI-Family and subsequent clinical outcomes). For the present research, alpha was set at 0.05, power at 0.8, and effect size at 0.4; yielding a sample size estimate of 250. This number is consistent with conclusions outlined in a recent review of tools for assessing patient-reported outcomes, which recommended that psychometric analyses include ≥200 individuals [18].

### Inclusion/exclusion criteria

Youth will be eligible to participate if they are 13 – 17 years old and have an age- and sex-specific BMI ≥97th percentile [19]. Including youth 13–17 years old will offer us diverse, detailed perceptions of motivational factors and lifestyle habits as opposed to involving younger youth (<13 years old). Parents will be eligible to participate if they self-identify as the primary caregiver of the youth referred for care. All participants must be able to read and communicate orally in English.

### Recruitment

Recruitment will take place at clinics, which family’s access through physician referral. As per clinic protocol, youth and families referred to the clinic will attend a group-based orientation session to meet clinic staff, receive information about available health services for weight management, and learn about active research studies. Subsequently, our team will liaise with clinic staff to retrieve contact information for families who expressed interest in learning more about our research. Once contacted, families’ study participation will be confirmed based on inclusion and exclusion criteria, and if included, research staff will complete informed consent and assent procedures. These steps are purposeful, designed to minimize perceptions of coercion on behalf of the research team, distinguish families’ clinical care from research, and mirror the recruitment process for our other ongoing multi-centre studies [20, 21].

This study will use a number of evidence-based strategies to recruit and retain families over the 12-month study period [22], including: *(i)* having a clear description of study expectations and commitments for families at the time of enrolment, *(ii)* corresponding with families using their preferred mode of contact (e.g., email, text message) to schedule research visits, *(iii)* disseminating tailored, 1-page study updates to share interim results with families to enhance study awareness, *(iv)* establishing a flexible schedule for booking research visits, *(v)* ensuring families understand the distinction between research and clinical care, *(vi)* working with clinical staff across both sites to share study updates and ensure mutual understandings, *(vii)* confirming families’ understanding of the value of their study participation, *(viii)* collaborating across sites to enhance recruitment and achieve recruitment targets, and *(ix)* offering gift cards (for families; ~$75 CDN value) and gift bags (for youth; ~$10 CDN value) as tokens of appreciation.

### Data collection

Each data collection time-point will require families to participate for approximately three hours. Assessment intervals are as follows (with varying assessment batteries to be completed at each time-point; see Table 1): *(i)* baseline (before families’ initiate multidisciplinary assessments that precede obesity management interventions), *(ii)* 6- and *(iii)* 12-months post-baseline assessment.

**Table 1:** RMI-Family study variables collected for families at baseline and 6- and 12-months post baseline

Variables		Baseline		6-months		12-months	
		*Youth*	*Parent*	*Youth*	*Parent*	*Youth*	*Parent*
RMI-FAMILY	1st interview *(administer & digitally record)* *[At enrollment] [n = 250]*	✓	✓				
	2nd interview *(administer & digitally record)* *[1 week after 1st interview] [n = 100]*	✓	✓				
DEMOGRAPHICS	Date of birth	✓	**✓**		✓ (If different parent)		✓ (If different parent)
	Gender	✓	✓		✓ (If different parent)		✓ (If different parent)
	Ethnicity	✓	✓		✓ (If different parent)		✓ (If different parent)
	Relationship status	✓	✓		✓ (If different parent)		✓ (If different parent)
	Household income		✓		✓ (If different parent)		✓ (If different parent)
	Parental education		✓		✓ (If different parent)		✓ (If different parent)
ANTHROPOMETRICS	Weight	✓	✓	✓	✓	✓	✓
	Height	✓	✓	✓	✓	✓	✓
	BMI	✓	✓	✓	✓	✓	✓
	BMI percentile	✓		✓		✓	
	BMI Z-score	✓		✓		✓	
MEDICAL	Obesity related co-morbidities	✓	✓	✓	✓	✓	✓
	Family history of chronic diseases	✓	✓		✓ ((If different parent)		✓ (If different parent)
	Family history of mental illness		✓		✓ (If different parent)		✓ (If different parent)
PSYCHOSOCIAL	Behavior Assessment System for Children – Third Edition (BASC-3)	✓	✓	✓	✓	✓	✓
	Behavior Rating Inventory of Executive Function – Second Edition (BRIEF 2)	✓	✓	✓	✓	✓	✓
	Emotion Regulation Questionnaire (ERQ)	✓	✓	✓	✓	✓	✓
	Coping with Children’s Negative Response Scale (CCNES)	✓		✓		✓	
	Marlowe-Crowne Social Desirability Scale *(MC-SDS)*	✓	✓	✓	✓	✓	✓
	Motivation to Change Ruler	✓	✓	✓	✓	✓	✓
LIFESTYLE	Nutrition intake *(24 h) (WEB-Q)*	✓		✓		✓	
	Dutch Eating Behaviour Questionnaire (DEBQ)	✓		✓		✓	
	Physical activity *(7 days with accelerometer)*	✓		✓		✓	
HEALTH SERVICES UTILIZATION	Total number of interactions: families & clinicians *(in-person and distance)*			✓	✓	✓	✓
	Number of interactions with specific clinicians			✓	✓	✓	✓
	Length of interaction			✓	✓	✓	✓
	Appointment changes/no shows			✓	✓	✓	✓
	Attrition			✓	✓	✓	✓

### The RMI-Family interview

#### Interviewer Training

Research team members will complete didactic and experiential training to administer the RMI-Family interview (see Additional file 1 for youth and parent interview guides). Interviewers will learn the operational definition of each domain included in the interview and will be trained to adopt an MI stance (i.e., collaborative, non-judgmental, curious) via a 1.5-day training session led by study investigators with expertise in MI, behavior change, and pediatric weight management. Following participation in training, interviewers will video-record at least four practice sessions (two with a simulated youth; two with a simulated parent) that will be submitted to and reviewed by trainers on our team who will provide constructive feedback and recommend additional practice, as needed.

#### Administration

At both study sites, research staff will have access to private rooms for administering the interview. Separate interviews will be conducted with youth and parents; other study data (e.g., questionnaires, dietary assessment) will be collected following the RMI-Family interview to minimize the impact of considering this information on participants’ self-reported motivation for treatment. All interviews will be audio recorded and random samples of interviews will be drawn periodically over the course of the study for review by trainers to assess interviewer fidelity and provide feedback to interviewers, and code interview data.

#### Scoring

The RMI-Family generates several measures of individual- and family-level motivation (see Additional file 2 for scoring template). Youth and parent interviews each yield measures of *Youth* and *Parent Motivation*, reflecting participants' perceptions of the extent to which they value the domains being assessed (e.g., physical activity), the extent to which their other family member (i.e., youth or parent) values these domains, and the degree to which they believe that changing their behavior in these domains will be difficult.

Interviews will also yield measures of *Within-Youth* and *Within-Parent Concordance* (the extent to which respondents perceive that their other family member values each domain to a similar extent as themselves) and *Family Concordance* (the extent to which youth’s self-reported value of each domain agrees with their family member’s perception of their value level). These five scores will be calculated for each domain individually and across domains; the latter yielding *Total Motivation, Total Within-Participant Concordance, and Total Family Concordance* scores. Finally, the *RMI-Family Total Score* will be calculated to represent the sum of scores across domains assessed on individual motivation, within-participant concordance, and family-based concordance.

An interviewer and an independent research team member will score the first 10% of interviews (*n* = 25/250), then score 10% of the remaining interviews (*n* = ~22/225) to establish *inter-rater reliability*. The two independent ratings of the same interviews will be compared using intra-class correlation coefficients (ICCs) to assess inter-rater agreement for youth, parent, and family (youth and parent) interviews. Discrepancies will be resolved twice; after interviews are scored initially, followed by the second scoring. Further re-training of interviewers in MI techniques may take place as required. Reliability will be considered acceptable when ICCs ≥0.75 [23].

#### Administration fidelity

To complement interviewer training at baseline, the *Motivational Interviewing Supervision and Training Scale* (MISTS [24]) will be used to provide a behavioral count of interviewers’ skills that are consistent with the principles of MI. Four independent team members (who did not administer the interviews being checked) will complete the MISTS for 10% of interviews conducted by each interviewer. These reviewers’ ratings will be correlated to establish agreement, with ICCs ≥0.75 considered as an acceptable level of fidelity [23].

#### Repeat administration

A random sub-sample (*n* = 100/500; equal numbers from each study site) of families will complete the RMI-Family a second time, which will allow us to determine the stability of the tool based on test-retest reliability. Families will complete the RMI-Family approximately one week after the first interview, but prior to initiating care.

### Demography, anthropometry, and clinical data

Demographic data will be collected for youth and parents, including family mailing address, date of birth, gender, relationship between youth and parent, ethnicity, and indices of socioeconomic status, including household income and parental education. Anthropometric data will be collected post-baseline interview and will include weight, height, BMI, BMI percentile (youth only), and BMI z-score (youth only). Existing equipment at both study sites will be used to collect this data based on protocols established by our team members [25, 26]. In addition, obesity-related co-morbidities and family history of chronic disease, including history of mental illness, will be assessed for youth and parents. For efficiency, data will be retrieved from medical records at the clinics; however, data accuracy and completeness will be confirmed in-person with families. Methodological rigour will be optimized when gathering these data by adhering to recommendations for medical record review research [27, 28].

### Psychosocial data

A range of questionnaires will be used to examine links between individual- and family-level motivation for change, psychosocial functioning, and qualities of the parent-child relationship. All measures will be completed at baseline, and 6- and 12-months post-baseline assessment.

Parents and youth will complete the *Behavior Assessment System for Children – Third Edition* (*BASC-3*; *Parent Rating Scales* [PRS] and *Self-Report of Personality [SRP]* [29], respectively) to provide information about youth internalizing behavior (e.g., anxiety, depression), externalizing behavior (e.g., aggression), and adaptive functioning (e.g., social skills). Parents and youth will also complete parent and self-report forms of the *Behavior Rating Inventory of Executive Function* [30–32] *– Second Edition* (*BRIEF 2* [33]) to assess youth behavioural regulation skills (e.g., inhibition, emotional control), metacognition skills (e.g., initiation, self-monitoring), and overall executive functioning skills. Youth and parent responses on the *BASC-3* and *BRIEF 2* are compared to those provided by a standardization sample of same-age youth and their parents, yielding *T*-scores that provide a relative measure of functioning in relation to age-based norms.

All participants will also complete the 10-item *Emotion Regulation Questionnaire* (ERQ [34]; *Adult* and *Child/Adolescent Version*) assessing their own emotion regulation skills. Scores on this measure are represented as the sum of participants’ responses when each item is rated on a scale of 1 (*strongly disagree*) to 7 (*strongly agree)*.

Parents will be administered the BASC-3’s *Parenting Relationship Questionnaire (PRQ* [35]*)*, which assesses parents’ perceptions of the quality of the parent-child relationship (e.g., relational frustration, communication). As above, parents’ responses on this measure are represented as *T*-scores, indexed against those provided by a sample of parents with same-age youth.

Parents and youth will be administered the *Coping with Children’s Negative Emotions Scale* (*CCNES* [36]; Parent and Adolescent forms, respectively) to provide insight into each informant’s perceptions of typical parental responses in times of stress or conflict (e.g., when the target youth gets angry because he/she can’t get something that he/she really wants). This measure requires participants to rate the likelihood of themselves/their parent displaying six possible responses to nine different scenarios, each reflecting a different response style (e.g., punitive, problem-focused, minimizing). Scores on this measure are generated by summing ratings within each response style category, providing an index of the response styles each parent is most to least likely to use.

In order to help validate responses from the RMI-Family interview, parents and youth will complete 10-point *Motivation to Change Rulers* [37] to conceptualize readiness to change nutrition and physical activity habits in general as well as the domains specific to the RMI-Family with scores ranging from 1 (*definitely not ready to change*) to 10 (*definitely ready to change*).

Parents and youth will complete the 33-item *Marlowe-Crowne Social Desirability Scale (MC-SDS* [38]*)*, which assesses the extent to which individuals tend to respond in a socially-sanctioned manner. Scores on this measure are generated by summing the ratings on each item.

### Nutrition and physical activity data

Nutrition intake will be measured in youth using the *Waterloo Eating Behaviour Questionnaire* (WEB-Q [39]), a 24-h retrospective web-based dietary assessment tool that lets participants choose from >800 foods and beverages [40]. Once selected, foods appear as photographic images to allow participants to indicate portion sizes. Finally, selections are presented on a meal summary page that can be reviewed and revised as necessary. Youth will also complete the *Dutch Eating Behavior Questionnaire (DEBQ* [39]*)* – *Emotional Eating Subscale* to provide information about their emotional state during times of eating. This 13-item measure requires participants to rate their desire to eat when experiencing each listed emotion (e.g., irritated, lonely, anxious).

Physical activity, sedentary behaviour, and sleep will be objectively measured over a 7-day period using ActiGraph wGT3X-BT accelerometers (*ActiGraph Corp.,* Pensacola, FL). Once accelerometers are returned to the research team, data will be downloaded and data reduction procedures will be conducted. As part of the data reduction, cut-points validated for this age group will be used to classify time spent awake into *(i)* sedentary, *(ii)* light-, *(iii)* moderate-, or *(iv)* vigorous-intensity physical activity [41].

### Health services data

To quantify health services utilization, research staff will work closely with clinical team members at both study sites to track families’ care. We will record the following details of all interactions between families and clinicians at 6- and 12-months post-baseline assessment: *(i)* total number of interactions between families and clinicians, both in-person and distance (e.g., telephone, email, Telehealth), *(ii)* number of interactions with specific clinicians (e.g., dietitian, exercise specialist, physician), *(iii)* mode of interaction (e.g., individual, group), *(iv)* length of each interaction, *(v)* number of scheduled appointment changes (e.g., no-shows), and *(vi)* program attrition (if applicable). When these data are either incomplete or ambiguous based on medical record reviews, electronic data from the study sites’ clinic appointment booking systems will be accessed.

### Data management and analyses

Research Electronic Data Capture (*REDCap*) and LabKey will be used for database management, platforms that will help our team to meet high standards of data integrity, security, and confidentiality. These web-based, password-protected systems will also enable document sharing and intra-team communication across sites, with leadership and coordination based in Edmonton. Hard copies of study data will be retained securely at each study site.

All data analyses will be completed using Statistical Analysis System (SAS) version 9.3 (SAS Institute Inc.; Cary, NC). Consistent with Objective 1 of this study, cross-sectional analyses will be performed to measure psychometric properties of the RMI-Family interview.

We will assess *internal consistency* by calculating Cronbach’s alpha coefficients for each of the five RMI-Family sub-scale scores and the RMI*-*Family Total Score. This step will provide information about how RMI-Family scores correlate with each other, thus reflecting the extent to which they assess the same construct. *Test-retest reliability* will be calculated for the sub-sample of families (*n* = 100) who completed the interview a second time before starting weight management treatment. Given that some variability in responding is anticipated, the RMI-Family (sub-scale and total scores) will be considered to have good reproducibility if ICCs ≥0.75 [23, 42]. *Convergent validity* will be determined by the degree to which data from the RMI-Family converges (as represented by Spearman correlations) with other data that are related conceptually. We will calculate the correlation between *Total Motivation* scores for both youth and parents, as well as RMI-Family *Total Scores*, with youth and parent *Motivation to Change Ruler* scores. *Concurrent validity* will be calculated by exploring the extent to which motivational and concordance data from the RMI-Family are related to measured lifestyle habits. RMI-Family sub-scale and total scores will be correlated with average *(i)* dietary intakes of total energy (kilocalories/day) and treat foods (servings/day) and *(ii)* physical activity (minutes/day of light-, moderate- and vigorous- intensity), sedentary behavior and sleep. *Predictive validity* will be assessed via longitudinal analyses examining the predictive value of the RMI-Family (Objective 2) for outcomes relevant to managing pediatric obesity at 6- and 12-months post-baseline assessment. We will apply repeated-measures modeling to examine the extent to which the RMI-Family predicts changes in *(i)* lifestyle habits (total energy [kcal/day]; treat foods [servings/day]; sedentary behaviour [minutes/day], light-, moderate-, and vigorous- intensity physical activity [minutes/day], and sleep [minutes/night]), *(ii)* weight management (youth BMI z-score), and *(iii)* health services utilization (total number of interactions between families and clinicians) with adjustment for baseline values and demography (e.g., gender, age). This approach will allow us to account for cluster effects related to differences between our two study sites, if applicable, and to explore any hierarchical effects related to assessing motivation at the individual- versus family-level.

We will also examine the role of several moderators in the relationships outlined above. Including these variables in our analyses will allow us to investigate whether links between RMI-Family scores, present functioning, and future outcomes vary depending on participant and family characteristics. Moderators will include youth demographic characteristics (e.g., age, gender), family factors (e.g., household income), youth social-emotional and executive functioning, quality of the parent-youth relationship, and parent and youth emotion regulation skills. Finally, consistent with objective 3, we will apply multivariable logistic regression to determine whether motivation and concordance constructs from the RMI-Family predict attrition at *(i)* 6- and *(ii)* 12-months post-baseline assessment with adjustment for covariates (e.g., gender, age, BMI z-score).

## Discussion

Our research will contribute to applied health services research that is conducted in real-world settings, using a novel approach that capitalizes on the conceptual strengths and evidence base of a family-centered care (FCC) approach, family systems theory (FST), and MI for pediatric weight management. The FCC approach to health care decision-making emphasizes the pivotal role of the family in the life of a child and acknowledges the diversity of each family unit [43]. This approach is consistent with the contemporary management of pediatric obesity in that clinicians are encouraged to adopt a consultative role and families are empowered to take an active role in their care [44]. The clinician-family relationship is characterized by mutual respect, building on strengths, flexibility, and support. Given the principal role of families in facilitating behavior change in youth with obesity, the RMI-Family assesses each family member individually alongside youth-parent concordance in term of motivation to change to gain a broader measure and understanding of motivational constructs.

Conceptualizing obesity within a family systems theory (FST) framework accounts for the dynamic family system [45] and the impact of the various components of the family system (e.g., individuals, dyads) on each other. With FST, families are viewed as interconnected, whole units with cause-and-effect interactions and feedback loops that evolve in response to changes in the family environment [46]. In the treatment setting, parents are required to examine their own attitudes, beliefs, and behaviours – and the impact of these factors on both the youth and family system, as formative steps that help families to break the inter-generational transmission of obesity. Consistent with FST, the RMI-Family interview is designed to explore similarities, differences, and interactions between youth and parents regarding motivational factors that influence their lifestyle habits.

MI is an effective treatment approach for facilitating change across a range of behaviours [14–16]. It is a directive, client-centred approach that helps individuals resolve ambivalence about making changes to unhealthy habits [13]. In applying MI, clinicians use a number of communication strategies with clients, including rolling with their resistance to change, understanding their motivations, listening actively, and working to empower them [47]. MI requires clinicians to embody an open, non-judgmental stance that allows them to meet clients at whatever point they are at in the change process. The spirit of MI has been described as collaborative, evocative, and honoring client autonomy; it is the spirit of MI that is relevant to this work. MI remains novel in the field of pediatric obesity management, with some clinical trials recently completed [48–50]. However, in current clinical practice, pre-intervention obesity assessments tend to measure desire for weight loss rather than motivation to change [51]. Families may offer socially-desirable responses to clinicians’ questions about motivation to change, and clinicians may want to enroll families in interventions believing that clinic attendance represents a high degree of motivation to change. We believe that administering the RMI-Family using the spirit of MI will allow us (and eventually clinicians in their everyday practices) to gain more accurate and informed insights into both families’ motivation to change lifestyle habits and youth-parent concordance.

### Potential impact and future directions

Success in managing pediatric obesity is associated with a high number of interactions between clinicians and families [52]. Unfortunately, declines in treatment adherence and attrition are commonplace in managing pediatric obesity [53–55], limiting the benefits families can derive from treatment and the extent to which treatment resources are optimized. Our research was designed to establish an innovative, psychometrically-sound tool that will increase clinicians’ and families’ understanding of the role that individual family members and characteristics of the family unit play in managing pediatric obesity. Not only have we designed our study to evaluate the effectiveness of the RMI-Family, but we will determine the role of motivational and concordance data in predicting clinical and health services outcomes over time.

Our research sets the stage for a number of clinical interventions, including the development of preparatory interventions to enhance motivational and concordance factors that are associated with improvements in clinically important outcomes. The RMI-Family may also be a useful tool for identifying motivational and concordance issues as they emerge over the course of treatment. Findings from this research will improve our understanding and collaboration between clinicians and family members and support the development of interventions that can enhance engagement, minimize attrition, and optimize treatment success. Since our research is based in primary care, it will be vital for our team to maximize its impact by disseminating study findings widely and testing the utility of the RMI-Family in community and primary care settings.

## Additional files

Additional file 1: The RMI-Family interview guides for youth and parent. Interviews administered to families (youth + parent). (DOCX 81 kb)

Additional file 2: The RMI-Family Scoring template. A scoring template to calculate individual- and family-level motivation and concordance. (DOCX 116 kb)

## Abbreviations

BASC: Behavior assessment system for children
BMI: Body mass index
BRIEF: Behaviour rating inventory of executive function
CCNES: Coping with children’s negative emotions scale
DEBQ: Dutch eating behavior questionnaire
ERQ: Emotion regulation questionnaire
FACES-IV: Family adaptability and cohesion evaluation scale IV
FCC: Family centered care
FST: Family systems theory
ICCs: Intraclass correlation coefficients
MC-SDS: Marlowe-crowne social desirability scale
MI: Motivational interviewing
MISTS: Motivational interviewing supervision and training scale
PMI: Parent motivation inventory
PRS: Parent rating scales
PRQ: Parenting relationship questionnaire
REDCap: Research electronic data capture
RMI-Family: Readiness and motivation interview for families
SAS: Statistical analysis system
SRP: Self report of personality
WEB-Q: Waterloo eating behaviour questionnaire

## Footnotes

1 Family members recruited along with youth are referred to as ‘parents’ throughout this paper for ease of reference; however, youth will be able to participate along with any adult who self-identifies as the youth’s primary caregiver (e.g., grandparent, guardian).
