# Supplementary material for: The readiness and motivation interview for families (RMI-Family) managing pediatric obesity: study protocol
Source: BMC Health Serv Res. 2017 Apr 11;17:261. doi: 10.1186/s12913-017-2201-8 (PMC5387327; doi:10.1186/s12913-017-2201-8)
Supplement: Supplementary file 1 — The RMI-Family interview guides for youth and parent. Interviews administered to families (youth + parent). (DOCX 81 kb) [file 12913_2017_2201_MOESM1_ESM.docx]

**Additional file 1.** The RMI-Family interview guides for youth and parents

**TEEN INTERVIEW**

*PREAMBLE:*

- This project is about learning how to help young people and their families have the best possible health. We believe that healthy bodies come in all shapes and sizes.
- We want to understand what is important to YOU about your physical activity and eating.
- The fun part of this interview is that after we talk about what YOU think we will ask you what you think your PARENT would say and what your ______________________ would say.
- We want you to feel you can say what’s really on your mind. So, everything you say in this interview is private! That means we won’t tell your parents or the treatment team what we talk about.
- Do you have any questions?

*WARM UP:*

- - How old are you? What grade are you in?
  - What do you do in your free time? Hobbies? Sports? Extracurricular activities?

1. **Physical Activity**

***We think of physical activity as movement that increases your heart rate, makes you feel warm, and a little out of breath. Examples include walking like you’re in a rush or late for class, riding a bike, or playing soccer. Do you do any physical activities?***

| ***YOU*** | ***YOUR PARENT*** | ***OTHER ADULT*** |
| --- | --- | --- |
| ***A1 – How important / how much do you care about your level of physical activity?***  **1** (not at all) **2** (a little) **3** (somewhat) **4** (very)  **5** (extremely) | ***A2 – How important / how much do you think your parent cares about your level of physical activity?***  **1** (not at all) **2** (a little) **3** (somewhat) **4** (very)  **5** (extremely) | ***A3 -- How important / how much do you think your _______________ cares about your level of physical activity?***  **1** (not at all) **2** (a little) **3** (somewhat) **4** (very)  **5** (extremely) |
| ***B1 – Do you think your level of physical activity is a problem?***  **1.** ____ not a problem  **2. ____** small problem  **3. ____** big problem | ***B2 – Does your parent think your level of physical activity is a problem?***  **1.** ____ not a problem  **2. ____** small problem  **3. ____** big problem | ***B3 – Does your __________ think your level of physical activity is a problem?***  **1.** ____ not a problem  **2. ____** small problem  **3. ____** big problem |
| ***C – If you decided to increase your physical activity, how hard would it be?***  **1** (not at all) **2** (a little) **3** (somewhat) **4** (very) **5** (extremely) | | |

1. **Screen Time**

***We think of screen time as any time you spend sitting while watching TV, movies, surfing the internet, texting, playing video games, etc. This does not include homework. What kinds of screen time activities do you participate in for fun?***

| ***YOU*** | ***YOUR PARENT*** | ***OTHER ADULT*** |
| --- | --- | --- |
| ***A1 – How important /how much do you care about the amount of time you spend in front of a screen?***  **1** (not at all) **2** (a little) **3** (somewhat) **4** (very)  **5** (extremely) | ***A2 – How important / how much does your parent care about the amount of time you spend in front of a screen?***  **1** (not at all) **2** (a little) **3** (somewhat) **4** (very)  **5** (extremely) | ***A3 – How important / how much does your________ care about the amount of time you spend in front of a screen?***  **1** (not at all) **2** (a little) **3** (somewhat) **4** (very)  **5** (extremely) |
| ***B1 – Do you think the amount of time you spend in front of a screen is a problem?***  **1.** ____ not a problem  **2. ____** small problem  **3. ____** big problem | ***B2 – Does your parent think the amount of time you spend in front of a screen is a problem?***  **1.** ____ not a problem  **2. ____** small problem  **3. ____** big problem | ***B3– Does your ­­­­­________ think the amount of time you spend in front of a screen is a problem?***  **1.** ____ not a problem  **2. ____** small problem  **3. ____** big problem |
| ***C – If you decided to decrease your screen time, how hard would it be?***  **1** (not at all) **2** (a little) **3** (somewhat) **4** (very) **5** (extremely) | | |

1. **Sleep**

***Tell me about your sleep – what time do you go to bed? What time do you get up? Do you ever wake up after you fall asleep? If so, is it hard to get back to sleep? Is there anything about the amount of sleep you get that you experience as a problem?***

| ***YOU*** | ***YOUR PARENT*** | ***OTHER ADULT*** |
| --- | --- | --- |
| ***A1 – How important is the amount of sleep that you get to you?***  **1** (not at all) **2** (a little) **3** (somewhat) **4** (very)  **5** (extremely) | ***A2 – How important is the amount of sleep that you get to your parent?***  **1** (not at all) **2** (a little) **3** (somewhat) **4** (very) **5** (extremely) | ***A3 – How important is the amount of sleep that you get to your ____________________?***  **1** (not at all) **2** (a little) **3** (somewhat) **4** (very)  **5** (extremely) |
| ***B1 – Do you think the amount of sleep you get is a problem?***  **1.** ____ not a problem  **2. ____** small problem  **3. ____** big problem | ***B2 – Does your parent think the amount of sleep you get is a problem?***  **1.** ____ not a problem  **2. ____** small problem  **3. ____** big problem | ***B3 – Does your ___________ think the amount of sleep you get is a problem?***  **1.** ____ not a problem  **2. ____** small problem  **3. ____** big problem |
| ***C – If you decided to increase/decrease the amount (circle their answer) of sleep that you get, how hard would it be?***  **1** (not at all) **2** (a little) **3** (somewhat) **4** (very) **5** (extremely) | | |

1. **Eating (“treat”) food**

***Sometimes we eat foods that we really like that are good for us, and sometimes we eat foods that we really like that are less nutritious for us. I like (1) pizza and pizza pockets, (2) muffins and chocolate bars, and (3) coca cola and energy drinks. What are some of your favourite “treat” foods?***

| ***YOU*** | ***YOUR PARENT*** | ***OTHER ADULT*** |
| --- | --- | --- |
| ***A1 – How important / how much do you care about the amount of “treat” food that you eat?***  **1** (not at all) **2** (a little) **3** (somewhat) **4** (very)  **5** (extremely) | ***A2 – How important / how much does your parent care about the amount of “treat” food that you eat?***  **1** (not at all) **2** (a little) **3** (somewhat) **4** (very)  **5** (extremely) | ***A3 -- How important / how much does your ___________ care about the amount of “treat food” that you eat?***  **1** (not at all) **2** (a little) **3** (somewhat) **4** (very)  **5** (extremely) |
| ***B1 – Do you think the amount of “treat” food you eat is a problem?***  **1.** ____ not a problem  **2. ____** small problem  **3. ____** big problem | ***B2 – Does your parent think that the amount of “treat” food that you eat is a problem?***  **1.** ____ not a problem  **2. ____** small problem  **3. ____** big problem | ***B3 – Does your _______ think that the amount of “treat” food that you eat is a problem?***  **1.** ____ not a problem  **2. ____** small problem  **3. ____** big problem |
| ***C – If you decided to decrease the amount of “treat” food that you eat, how hard would it be?***  **1** (not at all) **2** (a little) **3** (somewhat) **4** (very) **5** (extremely) | | |

1. **Overeating/Eating too much food**

***Sometimes we eat a lot of food and feel uncomfortably full, wish that we hadn’t eaten that much, or felt like we couldn’t stop eating. Does this ever happen for you? Can you describe a time when you felt you ate too much?***

| ***YOU*** | ***YOUR PARENT*** | ***OTHER ADULT*** |
| --- | --- | --- |
| ***A1 – How important / how much do you care about any overeating you may be doing?***  **1** (not at all) **2** (a little) **3** (somewhat) **4** (very)  **5** (extremely) | ***A2 – How important / how much does your parent care about any overeating you may be doing?***  **1** (not at all) **2** (a little) **3** (somewhat) **4** (very)  **5** (extremely) | ***A3 – How important / how much does your ________ care about any overeating you may be doing?***  **1** (not at all) **2** (a little) **3** (somewhat) **4** (very) **5** (extremely) |
| ***B1 – Do you think that overeating is a problem for you?***  **1.** ____ not a problem  **2. ____** small problem  **3. ____** big problem | ***B2 – Does your parent think that overeating is a problem for you?***  **1.** ____ not a problem  **2. ____** small problem  **3. ____** big problem | ***B3 – Does your _________ think that overeating is a problem for you?***  **1.** ____ not a problem  **2. ____** small problem  **3. ____** big problem |
| ***C – If you decided to decrease how often or how much you overeat, how hard would it be?***  **1** (not at all) **2** (a little) **3** (somewhat) **4** (very) **5** (extremely) | | |

1. **Eating when not hungry / Emotional eating**

***Sometimes we eat when we’re hungry, because our stomach is empty and our body needs food, and sometimes we eat for other reasons, like when we are bored, distracted, or sad. Do you ever eat when you’re not hungry but just feel like eating (e.g., noticing that food is gone, like a bag of chips)? Can you describe a time when you ate and weren’t hungry?***

*choose one depending on teen’s description of eating behavior* ***For the purposes of this question, we will call this “eating when not hungry” OR “emotional eating”.***

| ***YOU*** | ***YOUR PARENT*** | ***OTHER ADULT*** |
| --- | --- | --- |
| ***A1 – How important / how much do you care about any eating when not hungry/emotional eating that you may do?***  **1** (not at all) **2** (a little) **3** (somewhat) **4** (very)  **5** (extremely) | ***A2 – How important / how much does you parent care about any eating when not hungry/emotional eating that you may do?***  **1** (not at all) **2** (a little) **3** (somewhat) **4** (very)  **5** (extremely) | ***A3 – How important / how much does your _________ care about any eating when not hungry/emotional eating that you may do?***  **1** (not at all) **2** (a little) **3** (somewhat) **4** (very) **5** (extremely) |
| ***B1 – Do you think that eating when not hungry/emotional eating is a problem for you?***  **1.** ____ not a problem  **2. ____** small problem  **3. ____** big problem | ***B2 – Does your parent think that eating when not hungry/emotional eating is a problem for you?***  **1.** ____ not a problem  **2. ____** small problem  **3. ____** big problem | ***B3 – Does your ________ think that eating when not hungry/emotional eating is a problem for you?***  **1.** ____ not a problem  **2. ____** small problem  **3. ____** big problem |
| ***C – If you decided to decrease any eating when not hungry/ emotional eating you may do, how hard would it be?***  **1** (not at all) **2** (a little) **3** (somewhat) **4** (very) **5** (extremely) | | |

**PARENT INTERVIEW**

*PREAMBLE:*

- This project is about learning how to help teens and their families have the best possible health. We believe that healthy bodies come in all shapes and sizes.
- We want to understand how you feel about your teen’s physical activity and eating.
- After we ask you about what YOU think, we will also ask what you think your TEEN would say and what your ______________ would say.
- We want you to feel you can say what’s really on your mind. So, everything you say in this interview is private! That means we won’t tell your teen or your ______________ what you say.
- Do you have any questions?

*WARM UP:*

- - How many children do you have? Do you work outside the home?
  - What do you like to do in your spare time?

**1. Physical Activity**

***We think of physical activity as movement that increases your heart rate, makes you feel warm, and a little out of breath. Examples for your teen include walking when in a rush or late for class, riding a bike, or playing soccer. Does your teen enjoy any physical activities?***

| ***YOU*** | ***YOUR TEEN*** | ***OTHER ADULT*** |
| --- | --- | --- |
| ***A1 -- How important / much do you care about your teen’s level of physical activity?***  **1** (not at all) **2** (a little) **3** (somewhat) **4** (very)  **5** (extremely) | ***A2 -- How important / much do you think your teen cares about their level of physical activity?***  **1** (not at all) **2** (a little) **3** (somewhat) **4** (very) **5** (extremely) | ***A3 -- How important / how much does your ________ care about your teen’s level of physical activity?***  **1** (not at all) **2** (a little) **3** (somewhat) **4** (very)  **5** (extremely) |
| ***B1 -- Do you think your teen’s physical activity level is a problem?***  **1.** ____ not a problem  **2. ____** small problem  **3. ____** big problem | ***B2 – Does your teen think that his/her physical activity level is a problem?***  **1.** ____ not a problem  **2. ____** small problem  **3. ____** big problem | ***B3 – Does your ___________ think that his/her physical activity level is a problem?***  **1.** ____ not a problem  **2. ____** small problem  **3. ____** big problem |
| ***C. How much influence do you have over your teen’s level of physical activity?***  **1** (not at all) **2** (a little) **3** (somewhat) **4** (very) **5** (extremely) | | |
| ***D. If you were to decide to, how confident are you that you can change your teen’s level of physical activity?***  **1** (not at all) **2** (a little) **3** (somewhat) **4** (very) **5** (extremely) | | |

**2. Screen Time**

***We think of screen time as any time your teen spends sitting while watching TV, movies, surfing the internet, texting, playing video games, etc. This does not include homework. What kinds of screen time activities does your teen participate in for fun?***

| ***YOU*** | ***YOUR TEEN*** | ***OTHER ADULT*** |
| --- | --- | --- |
| ***A1 -- How important / much do you care about the amount of time your teen spends in front of a screen?***  **1** (not at all) **2** (a little) **3** (somewhat) **4** (very)  **5** (extremely) | ***A2 -- How important / much do you think your teen cares about the amount time he/she spends in front of a screen?***  **1** (not at all) **2** (a little) **3** (somewhat) **4** (very)  **5** (extremely) | ***A3 -- How important / how much does your _______ care about the amount of time your teen spends in front of a screen?***  **1** (not at all) **2** (a little) **3** (somewhat) **4** (very) **5** (extremely) |
| ***B1 -- Do you think the amount of time your teen spends in front of a screen is a problem?***  **1.** ____ not a problem  **2. ____** small problem  **3. ____** big problem | ***B2 – Does your teen think that the amount of time he/she spends in front of a screen is a problem?***  **1.** ____ not a problem  **2. ____** small problem  **3. ____** big problem | ***B3 – Does your _________ think that the amount of time he/she spends in front of a screen is a problem?***  **1.** ____ not a problem  **2. ____** small problem  **3. ____** big problem |
| ***C -- How much influence do you have over your teen’s screen time?***  **1** (not at all) **2** (a little) **3** (somewhat) **4** (very) **5** (extremely) | | |
| ***D -- If you were to decide to, how confident are you that you can change your teen’s screen time?***  **1** (not at all) **2** (a little) **3** (somewhat) **4** (very) **5** (extremely) | | |

1. **Sleep**

***Tell me about your teen’s sleep – what time do they go to bed? What time do they get up? Does your teen ever wake up after falling asleep? If so, is it hard for him/her to get back to sleep? Is there anything about the amount of sleep your teen gets that concerns you?***

| ***YOU*** | ***YOUR TEEN*** | ***OTHER ADULT*** |
| --- | --- | --- |
| ***A1 – How important / much do you care about the amount of sleep that your teen gets?***  **1** (not at all) **2** (a little) **3** (somewhat) **4** (very)  **5** (extremely) | ***A2 – How important / much do you think your teen cares about the amount of his/her sleep?***  **1** (not at all) **2** (a little) **3** (somewhat) **4** (very)  **5** (extremely) | ***A3 -- How important / how much does your _____ care about the amount of sleep that your teen gets?***  **1** (not at all) **2** (a little) **3** (somewhat) **4** (very) **5** (extremely) |
| ***B1 – Do you think that the amount of your teen’s sleep is a problem?***  **1.** ____ not a problem  **2. ____** small problem  **3. ____** big problem | ***B2 – Does your teen think that the amount of sleep he/she gets is a problem?***  **1.** ____ not a problem  **2. ____** small problem  **3. ____** big problem | ***B3 – Does your ______ think that the amount of sleep he/she gets is a problem?***  **1.** ____ not a problem  **2. ____** small problem  **3. ____** big problem |
| ***C – How much influence do you have over the amount of sleep your teen gets?***  **1** (not at all) **2** (a little) **3** (somewhat) **4** (very) **5** (extremely) | | |
| ***D. If you were to decide to, how confident are you that you can change the amount of sleep your teen gets?***  **1** (not at all) **2** (a little) **3** (somewhat) **4** (very) **5** (extremely) | | |

1. **Eating “treat” foods**

***Sometimes we eat foods that we really like and that are good for us, and sometimes we eat foods that we really like but that aren’t as good for us. What are some of your teen’s favourite “treat” foods?***

| ***YOU*** | ***YOUR TEEN*** | ***OTHER ADULT*** |
| --- | --- | --- |
| ***A1 -- How important / much do you care about the amount of “treat” food your teen eats?***  **1** (not at all) **2** (a little) **3** (somewhat) **4** (very)  **5** (extremely) | ***A2 -- How important / much do you think your teen cares about the amount of “treat” food she/he eats?***  **1** (not at all) **2** (a little) **3** (somewhat) **4** (very)  **5** (extremely) | ***A3 -- How important / how much does your ________ care about the amount of “treat food” that your teen eats?***  **1** (not at all) **2** (a little) **3** (somewhat) **4** (very) **5** (extremely) |
| ***B1 -- Do you think that the amount of “treat” food your teen eats is a problem?***  1. ____ not a problem  2. ____ small problem  3. ____ big problem | ***B2 – Does your teen think that the amount of “treat” food that he/she eats is a problem?***  1. ____ not a problem  2. ____ small problem  3. ____ big problem | ***B3 – Does your _________ think that the amount of “treat” food that he/she eats is a problem?***  1. ____ not a problem  2. ____ small problem  3. ____ big problem |
| ***C. How much influence do you have over how much “treat” food your teen eats?***  **1** (not at all) **2** (a little) **3** (somewhat) **4** (very) **5** (extremely) | | |
| ***D. If you were to decide to, how confident are you that you can change how much your teen eats “treat” food?***  **1** (not at all) **2** (a little) **3** (somewhat) **4** (very) **5** (extremely) | | |

1. **Overeating/Eating too much food**

***Sometimes we eat a lot of food and feel uncomfortably full, wish that we hadn’t eaten that much, or felt like we couldn’t stop eating. Does this happen for your teen? Can you describe a time when you felt your teen ate too much?***

| ***YOU*** | ***YOUR TEEN*** | ***OTHER ADULT*** |
| --- | --- | --- |
| ***A1 -- How important / much do you care about any overeating your teen may do?***  **1** (not at all) **2** (a little) **3** (somewhat) **4** (very)  **5** (extremely) | ***A2 -- How important / much do you think your teen cares about any overeating he/she may do?***  **1** (not at all) **2** (a little) **3** (somewhat) **4** (very)  **5** (extremely) | ***A3 -- How important / how much does your ________ care about any overeating your teen may do?***  **1** (not at all) **2** (a little) **3** (somewhat) **4** (very) **5** (extremely) |
| ***B1 -- Do you think that overeating is a problem for your teen?***  1. ____ not a problem  2. ____ small problem  3. ____ big problem | ***B2 – Does your teen think that overeating is a problem for him/her?***  1. ____ not a problem  2. ____ small problem  3. ____ big problem | ***B3 – Does your ________ think that overeating is a problem for him/her?***  1. ____ not a problem  2. ____ small problem  3. ____ big problem |
| ***C. How much influence do you have over any overeating your teen may do?***  **1** (not at all) **2** (a little) **3** (somewhat) **4** (very) **5** (extremely) | | |
| ***D. If you were to decide to, how confident are you that you can decrease any overeating your teen may do?***  **1** (not at all) **2** (a little) **3** (somewhat) **4** (very) **5** (extremely) | | |

1. **Eating when not hungry/Emotional eating**

***Does your teen ever eat when he/she isn’t hungry but just feels like eating, such as when he/she is bored, distracted, or sad? Can you describe a time when your teen ate and wasn’t hungry?*** *choose one depending on parent’s description of teen’s eating behavior* ***For the purposes of this question, we will call this “eating when not hungry” OR “emotional eating”.***

| ***YOUR TEEN*** | ***YOU*** | ***OTHER ADULT*** |
| --- | --- | --- |
| ***A1 – How important / much do you care about any eating when not hungry/emotional eating that your teen may do?***  **1** (not at all) **2** (a little) **3** (somewhat) **4** (very)  **5** (extremely) | ***A2 – How important / much do you think your teen cares about any eating when not hungry/emotional eating that she/he may do?***  **1** (not at all) **2** (a little) **3** (somewhat) **4** (very)  **5** (extremely) | ***A3 -- How important / how much does your _______ care about any eating when not hungry/emotional eating that your teen may do?***  **1** (not at all) **2** (a little) **3** (somewhat) **4** (very) **5** (extremely) |
| ***B1 – Do you think that eating when not hungry/emotional eating is a problem for your teen?***  **1.** ____ not a problem  **2. ____** small problem  **3. ____** big problem | ***B2 – Does your teen think that eating when not hungry/emotional eating is a problem for him/her?***  **1.** ____ not a problem  **2. ____** small problem  **3. ____** big problem | ***B3 – Does your ________ think that eating when not hungry/emotional eating is a problem for him/her?***  **1.** ____ not a problem  **2. ____** small problem  **3. ____** big problem |
| ***C – How much influence do you have over any eating when not hungry/emotional eating your teen may do?***  **1** (not at all) **2** (a little) **3** (somewhat) **4** (very) **5** (extremely) | | |
| ***D -- If you were to decide to, how confident are you that you can decrease your teen’s eating when not hungry/emotional eating?***  **1** (not at all) **2** (a little) **3** (somewhat) **4** (very) **5** (extremely) | | |
