# Supplementary material for: The readiness and motivation interview for families (RMI-Family) managing pediatric obesity: study protocol
Source: BMC Health Serv Res. 2017 Apr 11;17:261. doi: 10.1186/s12913-017-2201-8 (PMC5387327; doi:10.1186/s12913-017-2201-8)
Supplement: Supplementary file 2 — The RMI-Family Scoring template. A scoring template to calculate individual- and family-level motivation and concordance. (DOCX 116 kb) [file 12913_2017_2201_MOESM2_ESM.docx]

**Additional file 2**. The RMI-Family Scoring template

***Abbreviations and subscripts***:

Physical Activity = PA; Screen Time = ST; Treat Food = FT; Overeating = OE;

Eating Not Hungry =ENH

Examples: ***Ch*PA_B_** refers to Youth interview, Physical activity domain, question B.

***Par*OEc** refers to Parent interview, Overeating domain, question C

.

**1. Youth and Parent Motivation Sub-Scale Scores**

| Youth Motivation Score (sum) | Parent Motivation Score (sum) |
| --- | --- |
| ***Ch*PA_B_ *+ Ch*PA_C_ (reverse scored) *=***  ***Ch*ST_B_ *+ Ch*ST_C_ (reverse scored)  *=***  ***Ch*TF_B_ *+ Ch*TF_C_ (reverse scored) *=***  ***Ch*OE_B_ *+ Ch*OE_C_ (reverse scored) *=***  ***Ch*ENH_B_ *+ Ch*ENH (reverse scored) *=***  **Total Youth Motivation Sub-Scale Score**  **(Total score range: 10 – 50)** | ***Par*PA_B_ + *Par*PA_C_ + *Par*A_D_ =**  ***Par*ST_B_ + *Par*ST_C_ + *Par*ST_D_ =**  ***Par*TF_B_ + *Par*TF_C_ + *Par*TF_D_ =**  ***Par*OE_B_ + *Par*OE_C_ + *Par*OE_D_ =**  ***Par*ENH_B_ + *Par*ENH_C_ + *Par*ENH_D_ =**  **Total Parent Motivation Sub-Scale Score**  **(Total score range: 15 – 45)** |

**2a. Family Concordance Sub-Scale Scores**

| **Family (sum)** |
| --- |
| If ***Ch*PA_A_** = ***Par*PA_A_**, *Concordance Family* PA = 1; If ***Ch*PA_A_** ≠ ***Par*PA_A_**, *Concordance Family* PA = 0  +  Absolute value of difference between **[*Ch*PA_B_** and ***Par*PA_B_] (reverse scored)**  **PA Family Concordance Sub-Scale Score** |

*Note:* Total Family Concordance Sub-Scale Score is the sum of PA, ST, T, OE and ENH Family Concordance scores. Total score range: 5 – 25.

**2b. Youth and Parent Within Concordance Sub-Scale Scores**

| **Youth Within (sum)** |
| --- |
| If ***Ch*PA_A_** = ***Youth estimate of Par*PA_A_**, *Concordance Youth* *Within* PA = 1; If ***Ch*PA_A_** ≠ ***Youth estimate of Par*PA_A_**, *Concordance Youth Within* PA = 0  +  Absolute value of difference between **[*Ch*PA_B_** and ***Youth estimate of* *Par*PA_B_] (reverse scored)**  **PA Youth Within Concordance Sub-Scale Score** |

*Note*: Total Youth Within Concordance Sub-Scale Score is the sum of PA, ST, T, OE and ENH Youth Within Concordance scores. Total score range: 5 – 25.

| **Parent Within (sum)** |
| --- |
| If ***Par*PA_A_** = ***Parent estimate of Ch*PA_A_**, *Concordance Parent Within* PA = 1; If ***Par*PA_A_** ≠ ***Parent estimate of Ch*PA_A_**, *Concordance Parent Within* PA = 0  +  Absolute value of difference between **[*Par*PA_B_** and ***Parent estimate of* *Ch*PA_B_] (reverse scored)**  **PA Parent Within Concordance Sub-Scale Score** |

*Note*: Total Parent Within Concordance Sub-Scale Score is the sum of PA, ST, T, OE and ENH Parent Within Concordance scores. Total score range: 5 – 25.

1. **Total RMI-Family Total Score (sum)**

| **Total Youth Motivation Sub-Scale +**  **Total Parent Motivation Sub-Scale +**  **Total Family Concordance Sub-Scale +**  **Total Youth Within Concordance Sub-Scale +**  **Total Parent Within Concordance Sub-Scale**  **______________________________________________________________________________**  **Total RMI-Family Total Score (total score range: 35 – 170)** |
| --- |
